# The TMEM16A channel as a potential therapeutic target in vascular disease

**DOI:** 10.1097/MNH.0000000000000967

**Published:** 2024-01-08

**Authors:** Rumaitha Al-Hosni, Rachel Kaye, Catherine Seoyoun Choi, Paolo Tammaro

**Affiliations:** Department of Pharmacology, University of Oxford, Mansfield Road, Oxford, UK

**Keywords:** hypertension, pericyte, stroke, TMEM16A/anoctamin 1, vascular smooth muscle

## Abstract

**Purpose of review:**

The transmembrane protein 16A (TMEM16A) Ca^2+^-activated Cl^−^ channel constitutes a key depolarising mechanism in vascular smooth muscle and contractile pericytes, while in endothelial cells the channel is implicated in angiogenesis and in the response to vasoactive stimuli. Here, we offer a critical analysis of recent physiological investigations and consider the potential for targeting TMEM16A channels in vascular disease.

**Recent findings:**

Genetic deletion or pharmacological inhibition of TMEM16A channels in vascular smooth muscle decreases artery tone and lowers systemic blood pressure in rodent models. Inhibition of TMEM16A channels in cerebral cortical pericytes protects against ischemia-induced tissue damage and improves microvascular blood flow in rodent stroke models. In endothelial cells, the TMEM16A channel plays varied roles including modulation of cell division and control of vessel tone through spread of hyperpolarisation to the smooth muscle cells. Genetic studies implicate TMEM16A channels in human disease including systemic and pulmonary hypertension, stroke and Moyamoya disease.

**Summary:**

The TMEM16A channel regulates vascular function by controlling artery tone and capillary diameter as well as vessel formation and histology. Preclinical and clinical investigations are highlighting the potential for therapeutic exploitation of the channel in a range of maladaptive states of the (micro)circulation.

The transmembrane protein 16A (TMEM16A) Ca^2+^-activated Cl^−^ channels (CaCCs) are gated open in response to elevations in the intracellular free Ca^2+^ concentration ([Ca^2+^]_i_). Thus, TMEM16A channels couple cellular Ca^2+^ handling to excitability in a range of cell types including arterial smooth muscle and other vascular cell types [1,2].

Using electrophysiology, quantitative RT-PCR, and siRNA-based gene silencing, TMEM16A was first demonstrated to form CaCCs in pulmonary artery smooth muscle cells (PASMCs) [3], and later found to be expressed in other artery types including large conduit, small systemic, coronary, and cerebral arteries [4,5,6,7^▪^]. Functional TMEM16A channels have also been detected in cerebral, retinal and skeletal muscle contractile pericytes [5,8^▪▪^] and in endothelial cells (ECs) of some vascular beds [9^▪▪^,^10^]. Vascular TMEM16A channels represent an attractive potential target for a range of diseases of altered vessel tone. 

**Box 1:**
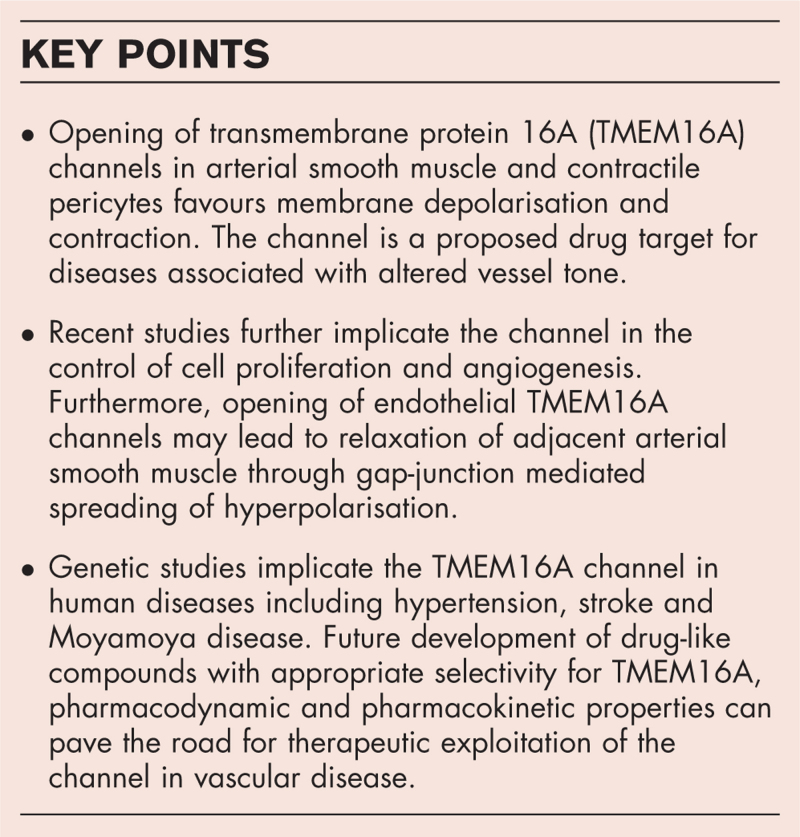
no caption available

## CHLORIDE HOMEOSTASIS IN VASCULAR CELLS

The extent of the TMEM16A channel's influence on cell function will depend on the direction and magnitude of the TMEM16A current (I_TMEM16A._). In the simplest approximation, I_TMEM16A_ is the product of the (i) number of TMEM16A channels expressed on the plasma membrane (N), (ii) single TMEM16A channel conductance (γ), (iii) electrochemical driving force – expressed as the difference between the cell membrane potential (*V*_m_) and the equilibrium (Nernst) potential for chloride (*E*_Cl_) - , and (iv) the channel open probability (P_o_) [11]. These factors are combined as:


(1) $$I_{TMEM16A}=N\cdot \gamma \cdot (V_{m}-E_{Cl})\cdot P_{o}$$

**FIGURE 1:**
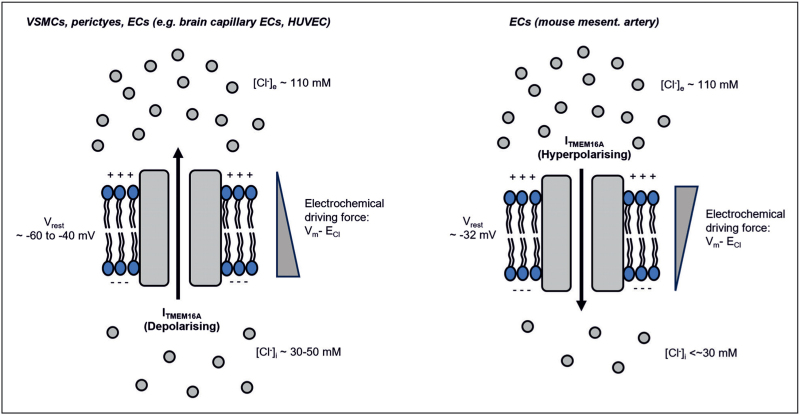
Direction of TMEM16A currents in vascular cells. The direction and magnitude of the TMEM16A current (*I*_TMEM16A_) in vascular cells determine its effect on the cell's membrane potential. In VSMCs, pericytes and some ECs (left panel), opening of TMEM16A channels leads to an efflux of Cl^−^, according to the Cl^−^ electrochemical gradient, and membrane depolarisation. In contrast, in some ECs, such as those of mouse mesenteric arteries, the lower [Cl^–^]_i_ means that the opening of TMEM16A channels will lead to Cl^−^ influx and membrane hyperpolarisation.

Thus, the net ionic current will be null when *V*_m_ equals *E*_Cl_ and will increase in magnitude in either direction as *V*_m_ deviates from *E*_Cl_ (Fig. 1).

**FIGURE 2:**
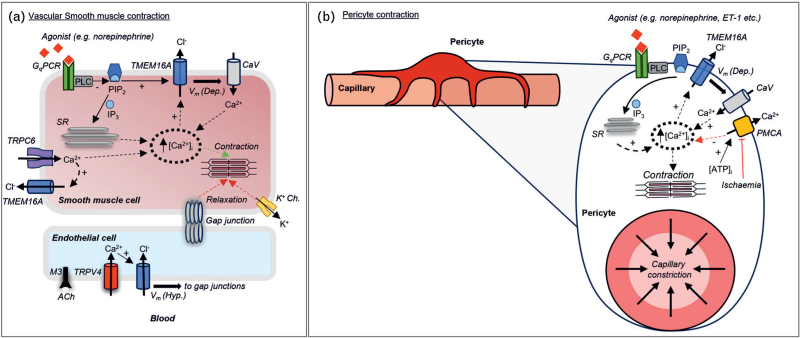
Roles for TMEM16A in VSMCs, ECs and contractile pericytes. (a) VSMCs and pericytes have a high Cl^−^ concentration [Cl^−^]_i_, produced by a series of active transport mechanisms in the plasma membrane (not shown). Agonists acting on G_q_PCRs stimulate the inositol tris-phosphate (IP_3_) pathway and Ca^2+^ release from the sarcoplasmic reticulum (SR). This initial rise in [Ca^2+^]_i_ promotes opening of TMEM16A channels, causing Cl^−^ efflux, *V*_m_ depolarisation and opening of CaV channels. The resulting further increase in [Ca^2+^]_i_ leads to cell contraction. Phospholipase C (PLC) activation that follows G_q_PCR stimulation, leads to depletion of plasmalemmal phosphatidylinositol 4,5-bisphosphate (PIP_2_). PIP_2_ is a stimulator of TMEM16A channel activity; thus, PIP_2_ depletion provides an antagonizing effect on the activation of the channel that follows IP_3_-mediated Ca^2+^ release. TMEM16A activation may also be coupled with Ca^2+^ entry via TRPC6 channels in VSMCs of some vascular beds. In some ECs, such as in mouse mesenteric arteries, acetylcholine (Ach) acting on muscarinic M3 receptors, leads to activation of the TRPV4 channel. The TRPV4-mediated Ca^2+^ entry activates TMEM16A channels leading to Cl^−^ influx; the resulting hyperpolarization can be transmitted through gap junctions to induce VSMCs relaxation. (b) Cerebral contractile pericytes (top panel: diagrammatic representation of a pericyte surrounding the cortical capillary; lower panel: representation of a pericyte soma with processes surrounding the capillary shown in cross section). The TMEM16A channel is activated following G_q_PCR stimulation, as in the VSMCs of the upstream arterioles. In ischemia, the reduction in intracellular ATP prevents Ca^2+^ extrusion through the plasma membrane Ca^2+^ ATPase (PMCA). The resulting increase in [Ca^2+^]_i_ favors pericyte contraction.

Both vascular smooth muscle cells (VSMCs) and pericytes have a high intracellular Cl^−^ concentration ([Cl^−^]_i_) (∼30 to ∼50 mM), produced by the plasma membrane Na^+^–K^+^–2Cl^–^ co-transporter NKCC1 and Cl^–^/HCO_3_^–^ exchanger AE2, while the physiological extracellular Cl^−^ concentration ([Cl^−^]_e_) is ∼110 mM [12–15]. Consequently, *E*_Cl_ spans from ∼–35 to −20 mV in these cells compared to a resting membrane potential (*V*_rest_) of ∼−60 to −40 mV. Opening of TMEM16A channels in contractile vascular cells therefore evokes Cl^−^ efflux (Eq. (1)) and depolarisation, which leads to activation of voltage-gated Ca^2+^ (CaV) channels, Ca^2+^ influx and contraction [12–14] (Figs. 1 and 2).

Consistently, TMEM16A expression and function is increased in arterial VSMCs of spontaneous hypertensive rats (SHRs) [16], including in renal [17] and coronary [4] artery VSMCs. Furthermore, vascular reactivity and systemic blood pressure are reduced in NKCC1 knockout mice [18], and NKCC1 expression and transport rate in VSMCs are increased in arteries obtained from a range of rat models of systemic hypertension [12]. These observations are consistent with the role of NKCC1 in increasing [Cl^−^]_i_ thus contributing to the generation of a depolarizing *E*_Cl_ in contractile vascular cells.

Although the depolarising role for TMEM16A in contractile vascular cells described above is well established, the role of TMEM16A in the control of *V*_m_ of ECs is not fully defined. The *V*_rest_ in ECs varies depending on vessel type, species and recording conditions. Reported *V*_rest_ values include (i) ∼−11 mV [19] and ∼−32 mV [9^▪▪^] for rat and mouse mesenteric artery ECs, respectively; (ii) ∼−27 mV for calf pulmonary artery ECs [20] and (iii) ∼−33 mV for human umbilical vein ECs (HUVEC) [21]. Furthermore, in cultured human aortic endothelial cells (HAECs), the recorded values of *V*_rest_ displayed large variability and were bimodally distributed with peaks at ∼−18 and −68 mV [22].

There is also some variability in the reported values for [Cl^−^]_i_ in different ECs, contributing to variability in *E*_Cl_. For example, in HUVEC [21] and HAECs [22], [Cl^−^]_i_ was ∼34 mM, a value comparable to that reported for VMSCs. In ECs of mouse mesenteric arteries, *E*_Cl_ was found to be hyperpolarised relative to *V*_rest_, (∼−32 mV, see above) indicative of [Cl^−^]_i_ of <∼35 mM [9^▪▪^] (Fig. 1). In contrast, in mouse brain capillary ECs, pharmacological inhibition or gene silencing with siRNA against TMEM16A caused *V*_m_ hyperpolarisation, suggesting that TMEM16A acts as a depolarising mechanism in these ECs; this implies that *E*_Cl_ is depolarised compared to *V*_rest_ in these cells [23]. Similarly, in the isolated endothelium of guinea pig mesenteric arteries, acetylcholine (Ach) activated a depolarising CaCC current which counteracted the endothelium-dependent hyperpolarization evoked by Ca^2+^-activated small (SK_Ca_) and intermediate (IK_Ca_) conductance K^+^ channels [24]. Additional early studies, conducted prior to the cloning of *TMEM16A,* demonstrated a role for a Cl^−^ current in the control of Ca^2+^ entry in HAECs; this observation is consistent with a depolarising role for CaCCs in ECs and subsequent CaV-dependent Ca^2+^ entry [25,26].

Collectively, the studies above suggested that [Cl^−^]_i_ varies in ECs of different vascular beds possibly resulting in TMEM16A currents of different magnitude and direction depending on vessel type. In the future, systematic assessments of [Cl^−^]_i_ in ECs of various vascular beds with methods such as fluorescence lifetime imaging (FLIM) of the Cl^−^ dye *N*-(ethoxycarbonylmethyl)-6-methoxyquinolinium bromide (MQAE; MQAE-FLIM) [27] or genetically encoded Cl^−^ reporters [28] may help to define *E*_Cl_ in ECs of various vascular beds and pathophysiological conditions.

The [Cl^−^]_i_ can also be dynamically regulated. For example, [Cl^−^]_i_ is decreased in HUVEC during stimulation with tumour necrosis factor-α (TNFα), an inflammatory cytokine [21]. We speculate that [Cl^−^]_i_ may also be modulated by (i) pharmacological treatments (e.g. with diuretics acting on NKCC1), (ii) different degrees of expression, inferred from RNAseq studies [29,30], of the active transport mechanisms (see above) involved in chloride uptake in vascular cells or (iii) circadian control of NKCC1 activity [31].

Deviations from normal plasma [Cl^−^]_e_, which will also impact *E*_Cl_, can occur in disease. Hypochloraemia ([Cl^−^]_e_ < 96 mM) is observed in patients with chronic cardiac failure and is associated with higher risk of mortality in patients with acute or chronic heart failure [32,33,34,35]. Hyperchloremia ([Cl^−^]_e_ > 110 mM), which accompanies a range of kidney disturbances, is an independent predictor for hypertension [36,37], and positively associated with increased mortality rate of hospitalised patients [34]. These conditions are expected to impact *E*_Cl_ and thus the magnitude of TMEM16A currents. For example, hyperchloremia may produce a hyperpolarising shift in *E*_Cl_ in VSMCs, and also have systemic effects such as favouring acidosis [38], collectively promoting vessel dilatation [39].

TMEM16A is permeable to HCO_3_^−^ in addition to Cl^−^[40]. The reversal potential for HCO_3_^−^ in vascular cells under normal conditions is expected to be ∼−20 mV, (i.e. depolarised from the resting potential of VSMC, pericytes and most ECs, see above). It remains to be established whether HCO_3_^−^ fluxes contribute to *V*_m_ depolarisation during TMEM16A opening in vascular cells and whether this effect may be altered during acid base disturbances such as metabolic or respiratory alkalosis/acidosis and associated alterations in plasma HCO_3_^−^ levels.

## CELLULAR PHYSIOLOGY OF TMEM16A IN VASCULAR SMOOTH MUSCLE CELLS

In VSMCs, activation of G_q_ protein-coupled receptors (G_q_PCRs) leads to an inositol 1,4,5-trisphosphate (IP_3_) mediated rise in [Ca^2+^]_i_; this, in addition to triggering some contraction, leads to the opening of TMEM16A channels and CaV-mediated Ca^2+^ entry. Thus, TMEM16A channels couple agonist binding and VSMC contraction (Fig. 2A). Consistently, knockout of the *Tmem16a* gene in mice [5,41^▪^] abolished the CaCC current in isolated VSMCs, strongly diminished the response of isolated arteries to G_q_PCR agonists and produced a decrease in systemic blood pressure [5].

Activation of G_q_PCRs also leads to depletion of plasmalemmal phosphatidylinositol 4,5-bisphosphate (PIP_2_). PIP_2_ is a positive modulator of TMEM16A activity in heterologous expression systems [1,42]. In contrast, a study reported that PIP_2_ inhibited CaCC currents in rat PASMCs [43]. We speculate the discrepancy may be attributed to potential, not yet identified ancillary subunits for TMEM16A or posttranslational modifications specifically occurring in PASMCs. Interestingly, PIP_2_ inhibition of CaCC currents in PASMCs was especially pronounced at depolarised *V*_m_[43], above the physiological range of *V*_m_ in PASMCs, although depolarised *V*_m_ may be favoured in pathological conditions. Whether CaCC inhibition in response to PIP_2_ may occur in VSMCs of other vascular beds and the physiological significance of this regulation, remain to be explored.

TMEM16A co-localises with IP_3_ receptors and CaV1.2 in mouse PASMCs [41^▪^]. This spatial arrangement may enable IP_3_-mediated Ca^2+^ release events, which are spatially localised and short-lasting, to translate into a sustained muscle response during G_q_PCR agonist stimulation. The positive feedback mechanism constituted by TMEM16A activation, *V*_m_ depolarisation and activation of CaV1.2 channels may underly this phenomenon.

In rat cerebral artery VSMCs, TMEM16A was found to co-localize with the transient receptor potential canonical 6 channel (TRPC6) [44]. Ca^2+^ entry through TRPC6 led to TMEM16A activation [44] (Fig. 2A). The proximity of TMEM16A and other Ca^2+^-activated channels, such as large conductance K^+^ (BKCa) channels, to Ca^2+^ release sites (including the ER, lysosomes and mitochondria) remains a point of interest. Elucidating this further may inform about the origin of specific cell responses such as spontaneous transient outward (STOCs) or inward (STIC) currents, known to involve activation of plasmalemmal BKCa channels and CaCCs, respectively. The opposing effects of these channels on the *V*_m_ of VSMCs play a role in the control of vascular tone.

TMEM16A was also found to interact with CaV channels in rat mesenteric and tail arteries, where experimentally-induced downregulation of TMEM16A lowered the expression and function of vascular L-type CaV channels [45,46]; however, the underlying mechanism and whether these regulations occur physiologically are undefined. Arguably, loss of TMEM16A may favour *V*_m_ repolarisation; this could interfere with the extent of recuperation from CaV inactivation and thus influence the magnitude of the whole-cell CaV current.

TMEM16A may reside in caveolae, cholesterol-rich plasmalemmal invaginations that form signalling hubs where channels and receptors tend to cluster. Caveolin-1 is a ubiquitous scaffolding protein in caveolae. Portal vein VSMCs obtained from caveolin-1 knockout mice had increased TMEM16A expression and current [47^▪^]. The mechanism by which Caveolin-1 modulates TMEM16A expression, however, remains to be defined.

## TMEM16A IN DISEASE OF VASCULAR SMOOTH MUSCLE CELLS

TMEM16A channels in VSMCs are modulated in rodent disease models. TMEM16A is expressed in rat coronary arteries and pharmacological inhibition of the channel attenuated agonist (5HT, U46619) induced artery constriction and increased coronary flow in Langendorff perfused rat heart preparations. TMEM16A mRNA expression and vascular response to the agonists was enhanced in coronary arteries obtained from SHR. The artery response was restored by inhibition of TMEM16A with T16Ainh-A01 and *N*-[(4-methoxy)-2-naphthyl]-5-nitroanthranilic acid (MONNA) [4]. However, the selectivity of these compounds has been questioned [48,49^▪^], complicating the interpretation of experimental results involving these agents.

Ischaemia-induced extracellular acidosis caused contraction of rat coronary arteries and this effect was reduced by inhibition of TMEM16A [50]. However, the inhibitors used (T16Ainh-A01, MONNA, niflumic acid (NFA), 5-nitro-2-(3-phenylpropylamino)-benzoate (NPPB) and benzobromarone) can interact with a range of targets [48,49^▪^]; for example benzobromarone activates BKCa channels and relaxes airway smooth muscle via this mechanism [51], and NFA is a generic chloride channel blocker, clinically used in the treatment of joint and muscular pain via inhibition of cyclooxygenase-2. Coronary arteries were also found to relax in response to chrysin, a natural flavonoid found in honey and medicinal plants, through inhibition of TMEM16A currents [52].

Small hindlimb and cerebral arteries from mice with high fat diet-induced type 2 diabetes (T2D) had increased TMEM16A mRNA and protein expression. Myogenic tone was enhanced in arteries obtained from these mice but prevented by genetic knockout of TMEM16A [53^▪^]. The expression and activity level of the Akt2 kinase protein was reduced in T2D mice arteries and siRNA-mediated knockdown of Akt2 increased arterial TMEM16A protein levels in nondiabetic mice. It was proposed that a decrease in Akt2 function stimulates TMEM16A expression in VSMCs, leading to vasoconstriction during T2D [53^▪^].

## TMEM16A IN CONTRACTILE PERICYTES

Contractile pericytes control microvascular blood flow in the brain and other vital organs. TMEM16A is expressed in rodent [5,8^▪▪^] and human [8^▪▪^] cortical cerebral pericytes, and modulates pericyte tone in response to physiological agonists [8^▪▪^]. During cortical cerebral ischaemia, metabolic impairment in pericytes leads to accumulation of [Ca^2+^]_i_, promoting activation of TMEM16A channels and capillary constriction [54,55] (Fig. 2B). In ischemic stroke, which may follow arterial thrombosis or embolism, reperfusion of capillaries downstream of the impaired artery may remain incomplete (termed ‘no-reflow’), even after the flow in the upstream artery is re-established [56,57]. Prolonged pericyte constriction and elevated [Ca^2+^]_i_ may cause persistent capillary occlusion and reduced regional blood flow [56,57], promoting no-reflow and ischemic damage. Furthermore, pericyte loss participates in the deterioration of integrity of the blood–brain barrier (BBB) function [58].

Intravenously administered CaCCInh-A01, a TMEM16A inhibitor, was shown to reduce the ischaemia-mediated BBB damage in mice, although the direct involvement of pericytes in this effect was not studied [59]. In a rodent stroke model, inhibition of TMEM16A with 2-(4-chloro-2-methylphenoxy)-*N*-[(2-methoxyphenyl)methylideneamino]-acetamide (Ani9) protected from microvascular ischaemic damage, dampened the rise in pericyte [Ca^2+^]_i_ and capillary constriction, collectively improving cerebrovascular reperfusion *in vivo*[8^▪▪^]. Selective knock-out of *Tmem16a* in pericytes in mice resulted in diminished capillary constriction in response to endothelin-1 (ET_1_), consistent with data involving Ani9 [8^▪▪^]. Mendelian Randomisation Analysis linked altered TMEM16A expression with poor patient recovery from ischemic stroke in human subjects [8^▪▪^].

The expression of TMEM16A in pericytes of other vital organs, such as the heart and kidneys, and their influence on the control of blood flow in these organs in health and disease remains to be established. TMEM16A is expressed in the hindlimb arteries in mice [53^▪^]; it is unknown whether TMEM16A also functions in pericytes, capillary sphincters or ECs of the microvasculature in skeletal muscle. It is also unestablished whether the channel is involved in the control of (i) muscular blood flow during acute exercise and whether the associated increase in microvascular shear stress may in turn influence TMEM16A channel activity [60] or (ii) capillary growth in response to chronic exercise [61]. Elucidating these involvements may shed light into the mechanisms of control of skeletal muscle blood flow in response to alterations in metabolic demand.

## TMEM16A IN ENDOTHELIAL CELLS

**FIGURE 3:**
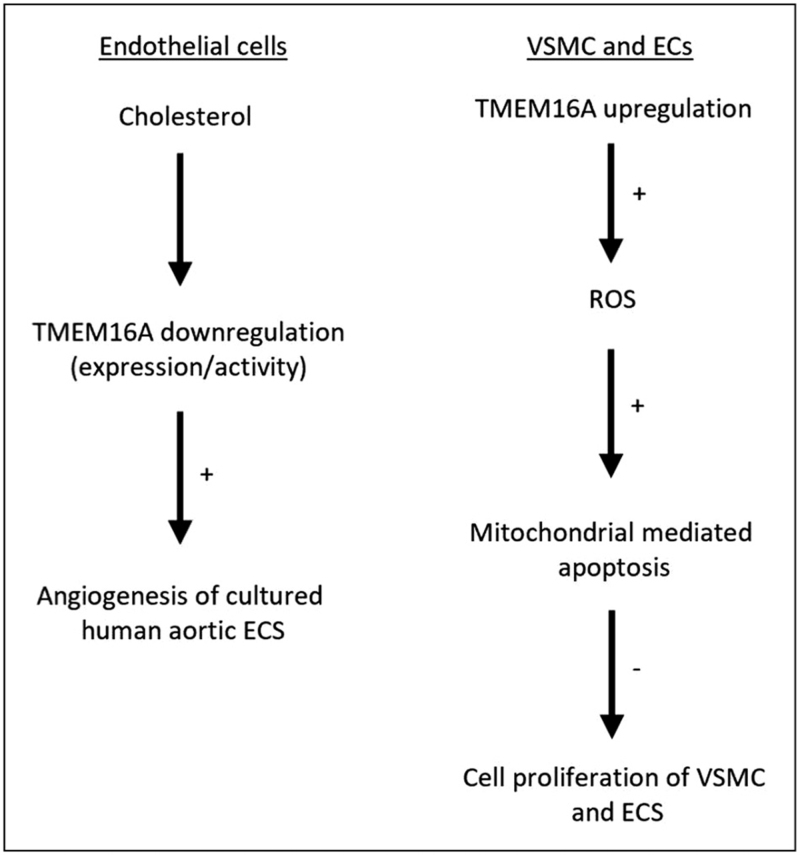
Pathways involved in epithelial and vascular smooth muscle cell proliferation. TMEM16A channel activity may be involved in the control of cell proliferation. Cholesterol may control the level of TMEM16A expression and suppresses TMEM16A channel activity. Mitochondrial TMEM16A activation may stimulate ROS production which in turn inhibits cell proliferation of ECs and VSMCs in some vascular beds, as detailed in the main text.

The contribution of TMEM16A to the *V*_m_ of ECs is not fully defined (see above, ‘Chloride homeostasis in vascular cells’). TMEM16A knockdown with siRNA or cholesterol-induced inhibition of the TMEM16A current stimulated proliferation and migration of HAECs [62] (Fig. 3). In angiotensin II (Ang II) induced hypertensive mice, TMEM16A protein expression in aortae was enhanced and TMEM16A knockout in ECs lowered systolic blood pressure (SBP) in these mice [10]. The underlying mechanism may involve TMEM16A-mediated reactive oxygen species generation via Nox2-containing NADPH oxidase [10]. In a separate investigation, overexpression of TMEM16A in human pulmonary ECs resulted in a decline in NO production in response to Ach [63]. Thus, TMEM16A in ECs is linked to regulation of diverse signalling pathways.

TMEM16A in ECs may have a role in the control of blood pressure. Constitutive knockout of *Tmem16a* in ECs in mice did not affect the basal contractility of isolated aortae or SBP [10]. Consistently, SBP was not affected in tamoxifen-inducible EC-specific *Tmem16a* knockout mice, but an increase in diastolic blood pressure (DBP) was observed [9^▪▪^]. The SBP is controlled by factors such as ejection fraction, aortic compliance, and the timing of wave reflection, while DBP is mainly determined by peripheral vascular resistance. The observations described above are consistent with the lack of effect of contractility of the aorta [10] and hypercontractile phenotype of small mesenteric arteries [9^▪▪^] in EC-specific TMEM16A knockout models. The loss of DBP was attributed to Ach-mediated activation of TRPV4 channels in mesenteric artery ECs resulting in Ca^2+^ influx and TMEM16A activation; this may promote membrane hyperpolarisation, which will transmit via gap junctions to VSMCs causing vasodilation [9^▪▪^] (Fig. 2A).

## ROLE OF TMEM16A IN VESSEL REMODELLING

Chloride channels have long been implicated in the control of proliferation of vascular cells [64,65]. More recently, TMEM16A was suggested to modulate tissue remodelling. Mice engineered to overexpress TMEM16A in VSMCs were protected from aortic [66] and cerebrovascular [67] remodelling during Ang II-induced hypertension. While the underlying mechanism is not fully defined, a suppression in autophagy and extracellular matrix deposition was reported in these mice [66,67].

TMEM16A expression is increased in aortic VSMCs during high salt-stimulated hypertension in mice. This was correlated with upregulation of pathways (such as the ESM1/VCAM-1 pathways) involved in VSMC inflammation [68]. TMEM16A inhibition reduced SBP of salt-sensitive hypertension in mice and alleviated vascular inflammation in these hypertensive mice [68].

High flow-induced pulmonary arterial hypertension induced in rats was associated with an increase in TMEM16A expression and alteration in the cell cycle of PASMCs. These cell cycle alterations were reversed by siRNA-mediated TMEM16A knockdown [69]. These observations suggest a role for TMEM16A in the regulation of cell cycle progression in PASMCs. The underlying mechanism, however, is not fully defined; it can be speculated that it may be secondary to the ability of TMEM16A to control *V*_m_ and Ca^2+^ handling and thus Ca^2+^/calmodulin-dependent signalling pathways involved in cell-cycle progression [70,71].

TMEM16A may also be expressed in the mitochondria of rat pulmonary artery ECs and in basilar artery smooth muscle cells [72,73]. In the mitochondria, TMEM16A was shown to activate cyclophilin D, a component of the mitochondrial permeability transition pore (mPTP), promoting pore opening. This results in the cleavage of caspase 9 and 3 triggering apoptosis. Reactive oxygen species (ROS) up-regulate this interaction by restricting the inhibitory effect of B-cell lymphoma 2 (bcl-2) on mPTP opening [73]. TMEM16A overexpression was suggested to inhibit cell proliferation by favouring mitochondria-dependent apoptosis via increased ROS in these ECs [72] and VSMCs [73] (Fig. 3).

## ROLE OF TMEM16A IN MOYAMOYA DISEASE

TMEM16A has been implicated in Moyamoya disease (MMD) which is characterised by progressive bilateral occlusion of the supraclinoid internal carotid artery and its main branches; this promotes formation of fine collateral networks, especially in correspondence to sites of occlusion [74]. MMD can be accompanied by ischaemic or haemorrhagic stroke, and cognitive impairment is often observed even among MMD patients who do not develop stroke [74]. *TMEM16A* gene variants, leading to missense mutations, have been linked to MMD [75^▪▪^]. Functionally, most of these variants result in gain-of-function defects (increased sensitivity of the channel to Ca^2+^). The carriers of these mutations displayed the typical characteristic of MMD (see above), but also aneurysm, stenosis and/or occlusion in the posterior circulation. Occluded carotid arteries from patients with MMD are characterised by neointimal lesions encompassing VSMC-like cells [75^▪▪^]. Increased proliferation and migration of VSMCs is a potential determinant of vessel abnormality in MMD [75^▪▪^]. The authors of this study suggested that enhanced TMEM16A currents may favour Cl^−^ efflux and associated Na^+^ and water loss and cell shrinkage, promoting VSMC migration [74]. The notion that TMEM16A overexpression is promoted in migration and proliferation of cancer and noncancer cells [76], further reinforces this proposition. It can be speculated that enhanced TMEM16A currents may also favour arterial and capillary constriction, contributing to ischaemic stroke propensity in MMD patients. Furthermore, gain-of-function of TMEM16A in ECs may dysregulate the extent of EC proliferation (see above, ‘TMEM16A in endothelial cells’) potentially contributing to MMD pathogenesis.

## IMPLICATIONS FOR THERAPY

Two population-based studies highlighted genetic *TMEM16A* variants associated with hypertension [4,77]. Mendelian Randomisation Analysis linked altered TMEM16A expression with poor patient recovery from ischaemic stroke [8^▪▪^]. Exome sequencing data for MMD patients have identified gain-of-function in *TMEM16A* as a cause for the disease [75^▪▪^].

The role of TMEM16A in systemic and pulmonary hypertension has been recently reviewed [12,78,79,80], and some key studies are described above. In general, mean arterial pressure in the long-term (hours to days) is mainly modulated by renal pressure natriuresis and diuresis, and associated modulation of cardiac preload and output. In the future, studies involving animal models of systemic hypertension or human subjects may benefit from assessment of renal function. This will include monitoring renal haemodynamics, plasma level of factors such as renin, Ang II, aldosterone, and ANP and electrolyte concentrations in the urine or possible proteinuria.

TMEM16A is involved in the control of cerebral pericyte tone [8^▪▪^]. It can be envisaged that TMEM16A inhibitors may be useful in alleviating ischaemia-induced capillary constriction or capillary constriction associated with other neurological disorders (e.g. traumatic brain/spinal cord injury, epilepsy, radiation necrosis, hydrocephalus, vascular dementia, and some forms of migraine) [56]. In these conditions, TMEM16A inhibition and associated capillary dilation may reduce the lack of energy, neurodegeneration and alteration of BBB function occurring in these disorders.

## CONCLUSION

A large body of evidence, spanning from studies involving animal models to pharmacological investigations and clinical studies, highlight TMEM16A as a depolarising influence in VSMCs of various artery beds and cerebral contractile pericytes. In ECs, upregulation of TMEM16A affects endothelial function, at least in part via reduction of NO release. Vascular TMEM16A also has a role in angiogenesis and vascular cell proliferation, and TMEM16A control of *V*_m_ in ECs may transmit to VSMCs to impact vessel tone. Studies involving human patients highlight the potential for TMEM16A as a new target for a range of diseases with a vascular component. While no therapeutic drugs acting on TMEM16A have yet reached use in clinical practice [79], there are exciting times ahead for exploring the therapeutic potential of this channel in a range of diseases of the (micro)circulation.

## Acknowledgements

*None.*

### Financial support and sponsorship

*Research in P.T.'s laboratory is supported by the British Heart Foundation (BHF) (PG/19/8/34168), the Biotechnology and Biological Sciences Research Council (BBSRC) (BB/T007664/1) and the Medical Research Council (MR/X010511/1).*

### Conflicts of interest

*There are no conflicts of interest.*

## REFERENCES AND RECOMMENDED READING

Papers of particular interest, published within the annual period of review, have been highlighted as:

▪ of special interest
▪▪ of outstanding interest
